# Intrapartum Ultrasound Assessment of Fetal Spine Position

**DOI:** 10.1155/2014/783598

**Published:** 2014-08-04

**Authors:** Salvatore Gizzo, Alessandra Andrisani, Marco Noventa, Giorgia Burul, Stefania Di Gangi, Omar Anis, Emanuele Ancona, Donato D'Antona, Giovanni Battista Nardelli, Guido Ambrosini

**Affiliations:** ^1^Department of Woman and Child Health, University of Padua, Via Giustiniani 3, 35128 Padova, Italy; ^2^Dipartimento di Salute della Donna e del Bambino, U.O.C. di Ginecologia e Ostetricia, Via Giustiniani 3, 35128 Padova, Italy

## Abstract

We investigated the role of foetal spine position in the first and second labour stages to determine the probability of OPP detection at birth and the related obstetrical implications. We conducted an observational-longitudinal cohort study on uncomplicated cephalic single foetus pregnant women at term. We evaluated the accuracy of ultrasound in predicting occiput position at birth, influence of fetal spine in occiput position during labour, labour trend, analgesia request, type of delivery, and indication to CS. The accuracy of the foetal spinal position to predict the occiput position at birth was high at the first labour stage. At the second labour stage, CS (40.3%) and operative vaginal deliveries (23.9%) occurred more frequently in OPP than in occiput anterior position (7% and 15.2%, resp.), especially in cases of the posterior spine. In concordant posterior positions labour length was greater than other ones, and analgesia request rate was 64.1% versus 14.7% for all the others. The assessment of spinal position could be useful in obstetrical management and counselling, both before and during labour. The detection of spinal position, more than OPP, is predictive of successful delivery. In concordant posterior positions, the labour length, analgesia request, operative delivery, and caesarean section rate are higher than in the other combination.

## 1. Introduction

The foetal head typically engages in the transverse diameter late in the third trimester and usually rotates to an occipitoanterior (OAP) or occipitoposterior (OPP) position. OPP occurs in 15–20% of women before labour at term [1].

Approximately 90–95% of these foetuses rotate during labour once the head reaches the pelvic floor [1, 2]. Thus, most of the OPP deliveries seem to arise as a consequence of a malrotation from the initial OAP or transverse position (OTP), rather than a persistent OPP. OPP incidence at birth ranges between 1 and 5% [2, 3].

Intrapartum ultrasound may improve the detection of fetal head position [4]. Although the identification of OPP before or during labour is not predictive of the same position at delivery, its early detection argues for a greater monitoring of the labour evolution [4, 5].

Blasi et al. [6] showed that the diagnostic sonographic accuracy of the foetal occiput position assessment at the second stage of labour had a sensitivity of 100%, specificity of 78%, positive predictive value (PPV) of 26%, and negative predictive value (NPV) of 100% to predict the same position at birth. Considering the foetal spinal position, ultrasound showed a sensitivity of 100%, specificity of 98%, PPV of 85%, and NPV of 100% [6].

Peregrine et al. [1] demonstrated that the foetal spine and occiput were often not concordant, but the posterior positioned spine was detected in nearly 14.5% of deliveries frequently associated with OPP [1]. Recent literature confirms that OPP represents an obstetric challenge because it is associated with an increased maternal foetal and neonatal morbidity, and its management is still debated [6].

In obstetrical practice, pregnant women with OPP foetuses present prolonged second stages of labour, higher rates of episiotomy, and severe perineal lacerations, mainly owing to the higher rates of instrumental delivery and increased risks of Caesarean section (CS) by nearly 4-fold [7].

The first aim of this study was to investigate the role of foetal spinal position in the first and second stages of labour in determining persistent OPP at birth. The second aim of the study was to investigate the implications of persistent OPP during labour in terms of the mode of delivery, length of labour, and analgesia request rate.

## 2. Patients and Methods

An observational study was conducted on pregnant women at term who delivered in the Gynaecological and Obstetric Clinic, Department of Woman and Child's Health of Padua University Hospital, between December 2011 and August 2013.

All patients were properly informed about the procedure and consented to the use of their data for this study by written consent, respecting their privacy (Italian Law 675/96).

After consulting the local ethical committee, our study was defined as exempt by the Institutional Review Board (IRB). Approval from the local IRB for the health sciences is not required for observational studies because the clinical management and/or surgical approach were not modified by the investigators. All patient data were made anonymous.

All women were consecutively enrolled by the researchers and carried out the ultrasound assessment at the first stage of labour, with or without the spontaneous rupture of membranes and a Bishop Score ≤7.

Inclusion criteria were as follows: age 18–40 years old, uncomplicated pregnancy, single foetus in cephalic presentation, normal foetal heart rate pattern status, and parity ≤3. We included also patients with history of third-trimester isolated oligohydramnios [8].

Exclusion criteria were as follows: history of uterine malformation, previous uterine surgery, pregnancies obtained by assisted reproductive techniques, suspicion of foetal malformation, intrauterine growth restriction, estimated foetal weight ≥4500 gr (calculated using ultrasound measurements by the Hadlock formula) [9], maternal fever of more than 38°C at admission, and incomplete obstetrical data about the trends of labour.

The stages of labour were established by the members of midwifery staff assisting the patients. The beginning of labour was defined by regular uterine contractions and changes in the cervical dilatation of more than 2 cm, according to the defined criteria [10]; the second stage was defined by attaining a full dilatation of the cervix.

For all of the patients, data were collected on the following: maternal age, gestational age, parity, type of labour (spontaneous or induced), length of the first and second stages of labour (in minutes), maternal request of epidural analgesia, type of delivery (spontaneous, operative, or Caesarean section), indications for caesarean section, neonatal weight (in grams), and length (in centimetres).

The ultrasound examination was performed by one of the researchers (Giorgia Burul) who had previously been trained for six months in the use of intrapartum ultrasounds. The researcher who performed intrapartum ultrasound examination was blinded to clinical examinations performed by midwifery or clinician during labour. The transabdominal (TA) scan was performed in the maternal supine position with a 3.5 MHz convex probe AB2-7-RS* (Voluson e6 compact-GE Healthcare, GE Medical Systems Ltd, Diagnostic Imaging/Ultrasound/Life Care Solutions, 71 Great North Road, Hatfield, Hertfordshire)*. As previously described [11–14], the landmarks depicting fetal occipital position (anterior, transverse, or posterior) were the fetal orbits for occiput posterior position, the midline cerebral echo for occiput transverse position, and cerebellum or occiput for occiput anterior position. The position of the foetal spine was determined by obtaining a transverse section of the foetal chest at the four-chamber view of the heart.

The positions of the spinal column and occiput were defined, as previously reported by Blasi et al. [6] and Akmal et al. [12], with the ultrasound findings for each foetus being reported on a clock-like chart divided into 12 sections, each representing 30°. The anterior position was determined if the occiput or spine was anterior (9.30–2.29), with other positions described as transverse right (8.30–9.29), transverse left (2.30–3.29), or posterior (3.30–8.29) [6]. At delivery, all foetal occiput positions were also recorded.

Occiput position and spinal column position were detected by TA ultrasound evaluation at the beginning of the labour (3 cm of cervical dilation) and at the second stage of labour (after the patient was diagnosed to be fully dilated); at birth, the occiput position was detected by clinical evaluation. When CS was performed before the complete cervical dilation, we documented occiput and spinal positions before the CS.

Midwifery and clinicians were blinded to ultrasound reports and to the aim of the study. They assisted the parturient according to our delivery room protocols and we collected information about labour from the final delivery report. Statistical analysis was performed by SPSS (IBM company, Chicago, IL, USA) software for Windows version 19, using parametric and nonparametric tests where appropriate. We performed the Kolmogorov-Smirnov test for the normality of the distribution. Continuous data were tested with *t*-tests, performing the ANOVA test when necessary, and categorical variables were tested with the *χ* ^2^ test or Fisher's exact test, where appropriate. The results obtained from the data collection were expressed in absolute numbers and percentages for discrete variables and in means ± standard deviations for continuous variables. Statistical significance was defined as *P* < 0.05.

## 3. Results

Among all of the pregnant women admitted to the delivery room of the Obstetric and Gynaecological Unit of Padua during the chosen time period, 256 patients were eligible for inclusion into the study.

Data about maternal age, gestational age, parity, type of labour, length of the first and the second stages of labour, maternal request of epidural analgesia, type of delivery, neonatal birth weight, neonatal length, and indications for Caesarean section were reported in Table 1.

Data about the occiput and spinal positions detected at the beginning and second stages of the labour and the occiput position at delivery were reported in Tables 2 and 3.

The diagnostic ultrasound accuracy of foetal occiput and spinal position in predicting the occiput position at birth was calculated only in the 210 patients who delivered vaginally.

The sensitivity of the OPP in predicting the same position at birth was 93.7% in the first stage of labour and 87.5% in the second stage of labour, with a specificity of 55.2% and 86.5%, respectively. The positive likelihood ratio was 2.09 in the first stage of labour and 6.48 in the second stage of labour, and the negative likelihood ratios were 0.11 and 0.14, respectively (Table 4).

The sensitivity of the posterior spinal position in predicting the same position at birth was 100% in the first stage of labour and 93.7% in the second stage of labour, and the specificities were 80% and 100%, respectively. The positive likelihood ratio was 41.7 in the first stage of labour and tended toward infinity at the second stage of labour, and the negative likelihood ratios were 0 and 0.06, respectively (Table 4).

Data about the type of delivery showed that 168 patients (65.6%) delivered vaginally without interventions, 42 (16.4%) delivered vaginally requiring interventions (operative delivery), and 46 (18%) delivered by CS.

From the correlations between occiput position at the second stage of labour and the resultant type of delivery, CS occurred in 40.3% of patients with OPP versus 38.9% of OTP and 7% of OAP patients (*P* < 0.05). On the contrary, no significant differences were detected between occiput position at the first stage of labour and the CS rate, despite OPP's showing a higher CS probability compared to the other positions.

Considering spinal position at second stage of labour, CS occurred in 63.4% of the posterior positioned, 15.4% of the transverse positioned, and 8% of the anterior positioned deliveries (*P* < 0.01).

In contrast to OPP, the CS rate showed a significant difference in relation to the spinal position at the first stage, with a rate of 57.4% when the spine was in the posterior position, 9.4% in the transverse position, and 8.2% in the anterior position (*P* < 0.01).

Operative vaginal delivery occurred in 23.9% of OPP detected at the second stage of labour versus 15.2% in OAP and none in OTP (*P* > 0.05). At the first stage of labour, no significant differences were detected among all of the occiput positions.

Regarding the spinal position detected at the second stage of labour, operative vaginal delivery occurred in 26.8% of the posterior positioned, versus 15.3% of the anterior positioned and 10.3% of the transverse positioned, deliveries (*P* < 0.01).

Regarding the spinal position detected at the second stage of labour, operative vaginal delivery occurred in 29.8% of patients with posterior spinal positions, in 16.3% with anterior positions, and in 12.5% with transverse positions (*P* < 0.01).

Data on the indications for CS and spinal position showed that all cases of dystocia (i.e., arrest of descent after two hours of active pushing) (13 patients) occurred when the spine was posterior, whereas only 39.4% (13 over 33 patients) of nonreassuring foetal hearts occurred in the same spinal position.

Data on patients who delivered vaginally (210 patients) showed that the length of the first stage of labour was 255.9 ± 119.6 minutes in OAP at birth, versus 439.7 ± 120.4 in OPP at birth (*P* < 0.001). Similarly, the length of the second stage of labour and the total length of labour in OPP at birth were significantly longer than OAP at birth: 98.13 ± 40.5 versus 56.4 ± 30.2 minutes and 537.8 ± 146.9 versus 312.2 ± 139.1 minutes, respectively (*P* < 0.01).

Among all patients, only 57 (23.3%) required epidural analgesia; in cases of concordant posterior position (occiput and spine) already at the first stage of labour, the rate of analgesia request was 64.1% versus 14.7% for all other combinations (*P* < 0.001).

An interesting datum was that, among the 16 patients (15 with posterior spine) who delivered vaginally a newborn presenting OPP at birth, 93.7% received epidural analgesia during labour; on the contrary, the remaining 26 posterior spinal positions (detected at the second stage of labour) who delivered by CS, 4 received analgesia in only 11.5% of cases (*P* < 0.001).

**Figure 1:**
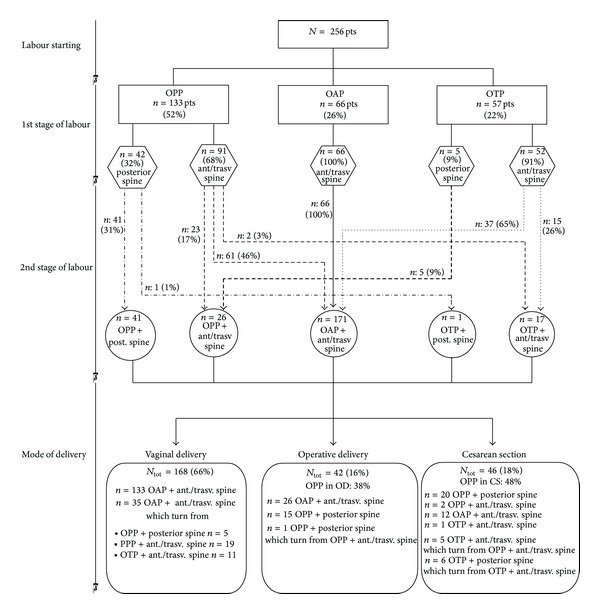
Synthetic flow diagram about fetal occiput and spine position at recruitment, during labour and at delivery (according to type of delivery). (i) OPP: occiput posterior position, (ii) OAP: occiput anterior position, (iii) OPT: occiput transverse position, (iv) posterior spine: foetal spinal column in posterior position, (v) ant./trasv. spine: foetal spinal column in anterior or transverse position (percentages are expressed each related to the proper cluster of population).

We provided to report data about fetal occiput and spine position at different stage of labour in the flow diagram (Figure 1).

## 4. Discussion and Conclusion

### 4.1. Main Findings

The foetal OPP during labour is associated with some unfavourable events, such as prolonged labour, need for assisted vaginal delivery, increased CS rate, and analgesia request rate [4, 6].

Although the OPP foetus usually rotates to OAP, this often occurs after many hours and efforts to address a painful, exhausting, and nonprogressing labour. The mother is at added risk for severe back pain, fatigue, and discouragement and is in greater need for emotional support. The new-born is at greater risk for a five-minute Apgar score <7, acidic cord blood gas concentrations, meconium-stained amniotic fluid, admission to neonatal intensive care, and longer hospitalization [15]. Thus, OPP poses clinical challenges in intrapartum care prevention, diagnosis, correction, supportive care, labour management, and delivery.

Currently, ultrasound evaluation at term represents the most accurate, easy, and reproducible tool for assessing both foetal position and pelvic-perineal findings [6, 15, 16]. The improvement of the ultrasound investigation during labour has led to the ability to distinguish patients whose pregnancies will result in spontaneous vaginal deliveries from those who will require operative vaginal delivery or CS for progression failure [13, 17, 18].

In women undergoing the induction of labour, preinduction sonographic determination of the occipital position, in addition to cervical length, is superior to the Bishop score in the prediction of induction outcome [19, 20]. Moreover, the sonographic detection of foetal head position is more accurate than vaginal examination both in the first and in the second stages of labour, particularly in OPP [21–23]. Despite these advantages, in the delivery room, ultrasound support still plays a secondary role with respect to clinical evaluation [1].

### 4.2. Strengths

Our strength points are the reported data about the different intrapartum outcomes (in homogeneous population) in relation to the spine position and the possible options that an early ultrasound detection of malposition could offer to women (more appropriate counseling, higher probability to benefit from fetal head manual rotation, and better management of labour and pain).

When OPP occurs, the assessment of the spinal position could be useful during the obstetrical decision-making process and the counselling of pregnant women before induction, or at the first stage of labour, especially when there are no indications for CS but poor chances of vaginal delivery.

### 4.3. Limitations

Our study had some limitations, due to the small sample size of the posterior spine and OPP cases and the performance of the ultrasounds by a single operator, even if the latter situation was necessary to eliminate interobserver bias. Another possible bias is linked to data about CS rate since in our unit (University Hospital) there is a high number of young resident gynecologists who resulted not skilled enough in performing intrapartum digital or manual rotation of the OPP in order to increase spontaneous deliveries and reduce CS and instrumental deliveries.

In our knowledge this study, differently from Blasi et al. [6], who focused only on intrapartum ultrasound diagnostic accuracy in predicting fetal spine and occiput position at different stages of labour, firstly investigated also the clinical impact of spine position in influencing the OPP persistence.

### 4.4. Interpretation

The importance of promoting intrapartum ultrasound examination is related to its high accuracy in predicting vaginal delivery and in facilitating the development of a decision-making strategy during labour [13]. According to this evidence, our data report that OPP at the second stage of labour is highly predictive of operative vaginal delivery or CS. Our study, according to Blasi et al. [6] and Peregrine et al. [1], reports that the concomitance between spine and occiput in the posterior position is more predictive than occiput alone in defining occiput position at birth. The posterior concomitance (occiput and spine), still in the first stage of labour, reveals a high accuracy in defining occiput position at birth and in predicting the labour trend.

Both the sensitivity and specificity of the spinal position are high in predicting the length of the labour and mode of delivery. The length of the labour, especially in the first stage, is increased in posterior spine when compared with the other positions. A spinal position different from the anterior position increases the risk of operative delivery and CS, even in the first labour stage, since, in our study, all cases of dystocia requiring CS occurred when the spine was posterior.

Many authors [3, 12, 24, 25] have tried to explain the exact mechanism that leads to an OPP at birth. However, the results conflict with each other. In fact the correct sonographic method (abdominal or perineal), the timing of the ultrasound study (the first or the second stage of labour), and the use of the parameters for foetal head position assessment are still controversial.

First, Blasi et al. [6] showed the importance of foetal spinal position in the intrapartum ultrasound assessment of the foetal position during the second stage of labour in predicting OPP at birth. They found higher OPP prevalence during the first and second stages of labour than expected. All OPP cases at delivery had the same position on ultrasound evaluation during the second stage of labour, in particular when they had a concordant spinal position. The authors [6] concluded that the concordant posterior position during the second stage of labour could be a useful indicator for predicting OPP at delivery.

In our sample, despite the fact that the concordant posterior position implies CS in 63.4% of cases, vaginal delivery was not impossible, even if 26.8% of cases required an intervention (increasing the risk of vaginal delivery complications) [26]. Our data show that the performance of epidural analgesia could play a beneficial role in promoting vaginal delivery when the occiput and spine are posterior, most likely facilitating the foetal pelvic progression and reducing, in this cohort, the rate of dystocia.

This concept may be based on the assumption that epidural analgesia induces pain relief and pelvic muscles relaxation, so it would reduce the resistance facilitating the foetal engagement to the maternal pelvis. The role of the concordant posterior position in increasing maternal pain is demonstrated by the high rate of requested analgesia. By the first stage of labour, 64.1% of deliveries in the posterior position versus 14.7% of all other combinations had requested analgesia.

According to our hypothesis, Lieberman et al. [27] reported that the OPP persisted at vaginal delivery in 12.9% of the epidural group versus 3.3% of the nonepidural group. However, our study, similarly to all the others reported in Literature, may be affected by the bias linked to a reduced maternal activity due to epidural analgesia administration. This could have a negative influence on the possible intrapartum fetal head rotation, sometimes facilitated by maternal mobilization during labour [15].

The increased rate in OPP occurrence among pregnant women receiving epidural analgesia may represent a mechanism by which epidural analgesia can be considered a risk factor for operative delivery and CS.

The confirmed beneficial effects of epidural analgesia [28] allow its administration within the OPP population, increasing the chances of vaginal delivery and reducing the intrapartum CS rate and its related complications [29, 30].

## 5. Conclusion

The assessment of the spinal position can be useful in the second stage of labour because it may help obstetricians to manage borderline intrapartum conditions, such as nonreassuring foetal heart rates, early signs of foetal distress, and maternal hyperpyrexia. On the contrary, the ultrasound assessment of OP alone before labour does not appear useful since foetuses with OPP at onset of labour seem to have both labour and delivery outcome similar to the OAP, particularly in case of anterior spine position [31]. The early intrapartum detection of OPP could anticipate and increase the possibilities of performing a fetal head manual rotation to OAP. In fact, some studies (despite none RCT) reported data about intrapartum digital or manual rotation of the OPP in order to increase spontaneous deliveries and reduce CS and instrumental deliveries [15]. Despite the fact that all the studies agreed with favorable findings of these procedures, only few maternity care practitioners use this procedure since the successful rotation depends on the experience and skill of the practitioner, on whether it is used selectively or routinely and on the timing of its performance [15].

## Figures and Tables

**Table 1:** Data about general maternal foetal features and labour characteristics.

Maternal, fetal, and labour features				
Variables		Number	Mean (±standard deviation)	Range
Maternal age (years)		256	31.06 (5.55)	17–42
Gestational age at birth (weeks)		256	39.41 (0.90)	39–41
Neonatal birth weight (gr)		256	3422.81 (389.63)	2300–4285
Neonatal length (cm)		256	49.37 (1.56)	46–53
Length of first stage of labour (minutes)		210∗	269.88 (128.96)	45–595
Length of second stage of labour (minutes)		210∗	59.55 (32.96)	10–165
Total length of labour (minutes)		210∗	329.43 (151.76)	60–725

Variables	Groups	Number (percentage)		

Parity	Nulliparous	163 (63.7)		
	Primiparous	66 (25.8)		
	Multiparous	27 (10.5)		

Type of labour	Spontaneous	182 (71.1)		
	Induced	74 (28.9)		

Epidural analgesia	Yes	57 (23.3)		
	No	199 (77.7)		

Type of delivery	Spontaneous	168 ( 65.6)		
	Operative	42 (16.4)		
	Cesarean section	46 (18.0)		

Indication for cesarean section	Dystocia	13 (5.1)		
	Non reassuring Fetal heart rate	33 (12.9)		

*Data about only patients who delivered through the vaginal route.

**Table 2:** Data about the occiput and spinal positions during the first and second stages of labour (for all patients) and at delivery (only in vaginal deliveries).

	Data about all patients				
		Number	Anterior number (%)	Posterior number (%)	Transverse (right or left) number (%)
First stage of labour	Occiput	256	66 (25.8)	133 (52.0)^§^	57 (22.3)
	Spine	256	49 (19.1)	47 (18.4)^#^	160 (62.5)
Second stage of labour	Occiput	256	171 (66.8)	67 (26.2)∗	18 (7.0)
	Spine	256	176 (68.8)	41 (16.0)∗∗	39 (15.2)

	Data about only patients who delivered through the vaginal route				
First stage of labour	Occiput	210	60 (28.6)	102 (48.6)	48 (22.8)
	Spine	210	45 (21.5)	20 (9.5)	145 (69.0)
Second stage of labour	Occiput	210	159 (75.8)	40 (19.0)	11 (5.2)
	Spine	210	162 (77.1)	15 (7.1)	33 (15.8)
Occiput at birth	—	210	194 (92.4)	16 (7.6)	—

^§^Of these, 71 cases changed position, 68 cases shifted to the occiput anterior position, and three cases shifted to the transverse position.

^#^Among the 47 cases of posterior spinal position, 42 (89%) showed a concordant occiput posterior position, whereas the remaining five cases showed a nonconcordant occiput position. The results in all cases were in the transverse position.

∗Of these, 62 already belonged to the occiput posterior position at the first stage of labour, and five changed into the occiput posterior position from the transverse position at first stage.

∗∗All of the 41 posterior spinal positions detected at the second stage of labour were in the same position at the first stage of labour. Only six posterior spinal positions at the first stage of labour changed into the other position, four cases changed to the anterior spinal position, and two cases changed to the transverse position.

**Table 3:** Detailed data about the posterior position (both occiput and spine) trends in the first and second stages of labour in terms of the concordant position and mode of delivery.

		I stage of labour (number)	II stage of labour (number)	Vaginal delivery (number)	Cesarean delivery (number)
Posterior position	Occiput	133	62	40	22
	Spine	47	41	15	26

Concordant posterior position	Occiput	42	41	15	20
	Spine				

**Table 4:** Estimation of the sensitivity, specificity, positive and negative predictive values (PPV and NPV), positive and negative likelihood ratios (LR+ and LR−), and their 95% CIs for the occiput and spinal position in the first and second stages of labour in predicting occiput posterior position at birth.

	Occiput posterior position (first stage of labour)		Spine posterior position (first stage of labour)	
	Value	95% CI
Sensitivity	0.937	0.904–0.970	1	0.923–1
Specificity	0.552	0.484–0.620	0.979	0.959–0.998
PPV	0.147	0.099–0.195	0.8	0.746–0.854
NPV	0.99	0.976–1	1	0.967–1
LR+	2.09		41.7	
LR−	0.11		0	

	Occiput posterior position (second stage of labour)		Spine posterior position (second stage of labour)	
	Value	95% CI	Value	95% CI

Sensitivity	0.875	0.830–0.920	0.937	0.904–0.970
Specificity	0.865	0.818–0.911	1	0.954–1
PPV	0.35	0.285–0.414	1	0.971–1
NPV	0.988	0.973–1	0.994	0.983–1
LR+	6.48		Infinity	
LR−	0.14		0.06	

*95% CI estimated by the Wilson method and by the binomial exact test, when necessary.
